# Treadmill running induces remodeling of the infrapatellar fat pad in an intensity-dependent manner

**DOI:** 10.1186/s13018-021-02501-7

**Published:** 2021-06-01

**Authors:** Ni Zeng, Tao Liao, Xin-Yuan Chen, Zhi-Peng Yan, Jie-Ting Li, Guo-Xin Ni

**Affiliations:** 1 Department of Rehabilitation Medicine, The First Affiliated Hospital of Fujian Medical University, Fuzhou, People’s Republic of China; 2 School of Sport Medicine and Rehabilitation, Beijing Sport University, Beijing, People’s Republic of China

**Keywords:** Infrapatellar fat pad, Treadmill running, Fibrosis, Remodeling, Inflammation

## Abstract

**Objective:**

To investigate the response of the infrapatellar fat pad (IFP) to running at different intensities and further explore the underlying mechanisms of these responses under different running-induced loadings.

**Methods:**

Animals were randomly assigned into the sedentary (SED), low-intensity running (LIR), medium-intensity running (MIR), and high-intensity running (HIR) groups. The rats in the LIR, MIR, and HIR groups were subjected to an 8-week treadmill running protocol. In each group, the IFP was examined at the baseline and at the 8th week to perform histomorphology, immunohistochemistry, and mRNA expression analyses.

**Results:**

Compared with LIR and MIR, HIR for 8 weeks led to a substantial increase in the surface cellularity (1.67 ± 1.15), fibrosis (1.29 ± 0.36), and vascularity (33.31 ± 8.43) of the IFP but did not increase IFP inflammation or M1 macrophage polarization. Low-to-medium-intensity running resulted in unchanged or decreased fibrosis, vascularity, and surface cellularity in the IFP compared to those of the SED group. Furthermore, serum leptin and visfatin levels were significantly lower in the LIR and MIR groups than in the SED group or the HIR group (*P* < 0.05).

**Conclusion:**

The effect of running on IFP remodeling was intensity dependent. In contrast to LIR and MIR, HIR increased the fibrosis and vascularity of the IFP. HIR-induced IFP fibrosis was probably due to mechanical stress, rather than pathological proinflammatory M1/M2 polarization.

## Introduction

The infrapatellar fat pad (IFP) is an intracapsular but extrasynovial structure located below the patella and adjacent to the synovial membrane [1]. This structure serves as a cushion between the patellar tendon and the anterior tibial plateau, thus protecting the knee joint from mechanical damage [2]. The IFP not only provides shock absorption and support for the knee joint but also secretes various cytokines and adipokines to interact with other joint tissues [3]. Recently, there has been increasing interest and awareness of the importance of the IFP in the pathogenic processes involved in knee osteoarthritis (KOA) [4]. In patients with KOA, compared with the subcutaneous adipose tissue (SAT), the IFP exhibited a more inflammatory phenotype and secreted higher levels of cytokines and adipokines, such as adiponectin, interleukin-6 (IL-6), tumor necrosis factor (TNF), and visfatin [5, 6]. Furthermore, contrary to that from non-OA controls, the IFP from patients with KOA showed pathological manifestations, such as fibrosis, vascularization, and inflammatory infiltrates [6].

Although accumulating evidence indicates that the IFP contributes to KOA by modulating the pro- or anti-inflammatory phenotype [3], its biological response to mechanical loading in the healthy knee is poorly understood. Mechanical factors have long been implicated in the etiology of OA [7]. An increased load on the knee may result in a significantly higher pressure inside the IFP at the extremes of extension and knee flexion [8]. More importantly, some aspects of IFP pathology, such as trauma-related fibrosis, calcification, and impingement, are associated with knee pain and are often referred to as Hoffa's disease [9].

Running is an excellent activity to promote general health and well-being. However, its effects on joint health appear to differ depending on the intensit y[10].. High-intensity running may induce a high degree of running-induced loading, which can be harmful to the joint once the magnitude exceeds the physiological tolerance level for an individual [7, 10, 11]. Currently, OA is regarded as a whole joint disease [12]. Using a rat model, we previously demonstrated that treadmill running at low-to-medium intensity maintained the homeostasis of cartilage and subchondral bone, whereas high-intensity running caused cartilage degradation and changed the organization, composition, and mechanical properties of subchondral bone [2, 13]. We found only one study in the literature that examined the effect of running on the IFP [9]. With a rat model, running was found to transiently enhance IFP inflammation, activate macrophages, and induce fibrosis, suggesting a physiological role for inflammation in load-induced IFP remodeling in young healthy knees [9]. However, a single running program was applied in this study. In this regard, the present study aimed to investigate the response of the IFP to running at different intensities and further explore the underlying mechanisms of the responses of the IFP under different running-induced loadings. We hypothesized that running may lead to an intensity-dependent effect on IFP remodeling and inflammation.

## Material and methods

### Animals

This study was approved by the Animal Ethics Committee of Fujian Medical University. Thirty male Sprague-Dawley rats at 8 weeks of age, 200–220 g in weight, were randomly and evenly assigned to one of five groups as follows: the (1) baseline, (2) sedentary (SED) group, (3) low-intensity running (LIR) group, (4) medium-intensity running (MIR) group, and (5) high-intensity running (HIR) group. The animals were housed under a 12 h/12 h light/dark cycle with food and water available ad libitum.

### Exercise protocols

The animals in all exercise groups were first accustomed to exercise for 1 week by running on a treadmill at a speed of 10 m/min for 30 min/day. Subsequently, according to previously described running protocols [2], the animals in the HIR group, LIR group, and MIR group ran on a motor-driven treadmill designed for rodents once a day, 5 days a week for 8 weeks. The speed and inclination were determined according to the following schema: LIR: 15.2 m/min with a 0° of inclination for 60 min, MIR: 19.3 m/min with a 5° of inclination for 60 min, and HIR: 26.8 m/min with a 10° of inclination for 60 min. The animals in the SED group were maintained in a sedentary state.

The animals in each group were euthanized under anesthesia by cervical dislocation either on the 1st day of the formal experiment (baseline) or the 8th week. Their IFPs from both sides were collected; the right IFP was used for histological assessment, and the left IFP was used for gene expression analysis.

### Histological assessment

Paraffin-embedded samples were used to prepare 4-μm-thick sections that were then stained with hematoxylin-eosin (HE) or Masson for histological assessment. The HE-stained sections of the IFP were used to determine the number and size of adipocytes, cellularity, number of crown-like structures per 1000 adipocytes, and vascularization, whereas the Masson-stained sections were used to assess the amount of collagen (fibrosis) in the IFP. The number and size of adipocytes in the IFP were determined using Image-pro plus 6.0 (Media Cybernetics, Inc., Rockville, MD, USA). The number of crown-like structures (a structure formed by monocytes/ macrophages localized in the periphery of degenerating adipocytes that is associated with the inflammation of adipose tissue) per 1000 adipocytes was evaluated to reflect the inflammatory state of the IFP according to previously described criteria [14]. Vascularization was evaluated in the HE-stained tissue sections; the number of vessels was counted in a whole slice, and four slices were counted for each sample to obtain the averaged value. The % area of fibrosis was measured using ImageJ software (National Institutes of Health, Bethesda, MD, USA). Additionally, IFP cellularity and fibrosis score were semiquantitatively evaluated according to previously described criteria [15].

### Immunohistochemical assessment

Sections were stained with CD86 and CD206 antibodies for the immunohistochemistry (IHC) evaluation and assessment of the phenotypes of macrophages that infiltrated into the IFP. Images were analyzed using Image-pro plus 6.0. Positive staining for CD86 indicated the M1 macrophage phenotype, and positive staining for CD206 indicated the presence of M2 macrophages.

### Serum adipokine measurements

After blood samples were kept at room temperature for 1 h, the serum was collected after centrifugation at 300 × *g* for 15 min and stored at − 80 °C until analysis. Serum levels of leptin and visfatin were detected by enzyme-linked immunosorbent assay (ELISA) according to the manufacturer’s instructions (Elabscience Biotechnology Co., Ltd., Wuhan, China).

### Quantitative real-time polymerase chain reaction (qRT-PCR)

Messenger RNA (mRNA) levels of PPARg, ATGL, LPL, SFRP2, HOXC9, IL-6, and MCP-1 were quantitated by quantitative polymerase chain reaction (qPCR) using a Real-Time PCR Detection System (Applied Biosystems, Stepone plus, USA). The PCR primer sequences are listed in Table 1. Fold changes (x-fold) in gene expression levels were calculated by the 2ct method.

**Table 1:** Primer sequences used for quantitative real-time polymerase chain reaction (RT-PCR)

Primer	Forward	Reverse (5′-3′)	Product size (bp)
GAPDH	CTGGAGAAACCTGCCAAGTATG	GGTGGAAGAATGGGAGTTGCT	138
PPARg	GTCTCACAATGCCATCAGGTTT	AGGGGGGTGATATGTTTGAACT	285
ATGL	GAACCGAAAGACCTGATGACCAC	CAGGCAGCCACTCCAACAAA	133
LPL	ATCAACAAGGTCAGAGCCAAGA	ATGTCCACCTCCGTGTAAATCA	248
Sfrp2	GTTCCTGTGCTCGCTCTTCG	CGTTGTCATCCTCGTTCTTAGTTT	272
Hoxc9	CAGCAAGCACAAAGAGGAGAAG	GGGCAGGGTTTAGGATTGTTC	292
IL-6	AAGCCAGAGTCATTCAGAGCAA	GTCTTGGTCCTTAGCCACTCCT	154
MCP-1	CCAATGAGTCGGCTGGAGAAC	GAAGTGCTTGAGGTGGTTGTGG	287

### Statistical analysis

Data were statistically analyzed using SPSS 23.0 software. All experimental data are expressed as the mean ± standard deviation. Differences between multiple groups were determined statistically by one-way ANOVA followed by a homogeneity test of variance. Post hoc LSD or Kruskal-Wallis H tests were used for multiple comparisons. *P* < 0.05 was considered to be statistically significant.

## Results

### Body weight

**Fig. 1:**
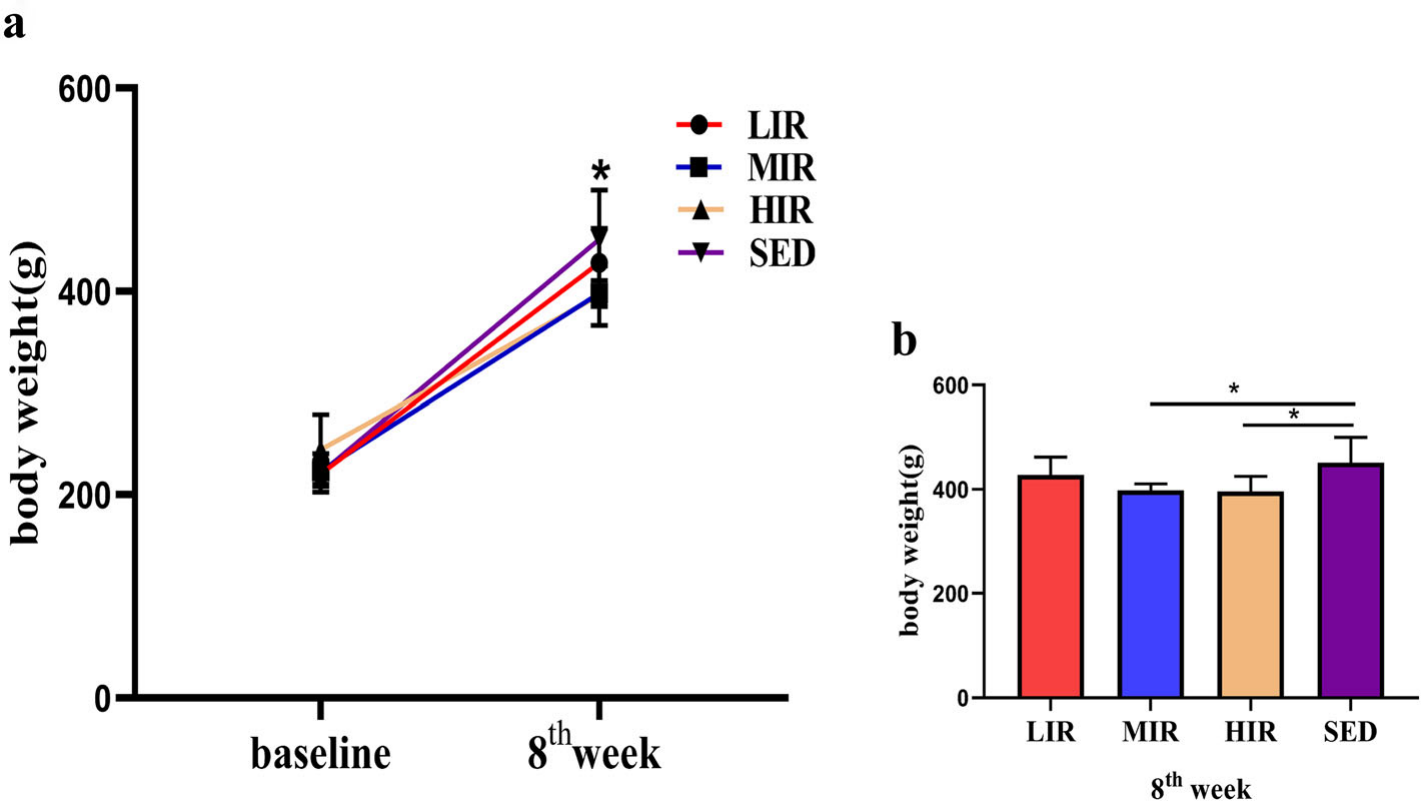
Body weight in the four groups. **a** body weight at the baseline and at the 8th week in each study group. * *P* < 0.05 compared to the body weight at the baseline in all groups. **b** Body weight at the 8th week in each study group; *P < 0.05

For each group, body weight at the 8th week was significantly higher than that at baseline. Although the body weight at baseline did not differ significantly among the four groups, both the MIR and HIR groups had a significantly lower body weight at the 8th week than the SED group (*P* < 0.05) (Fig. 1).

### Serum levels of leptin and visfatin

**Fig. 2:**
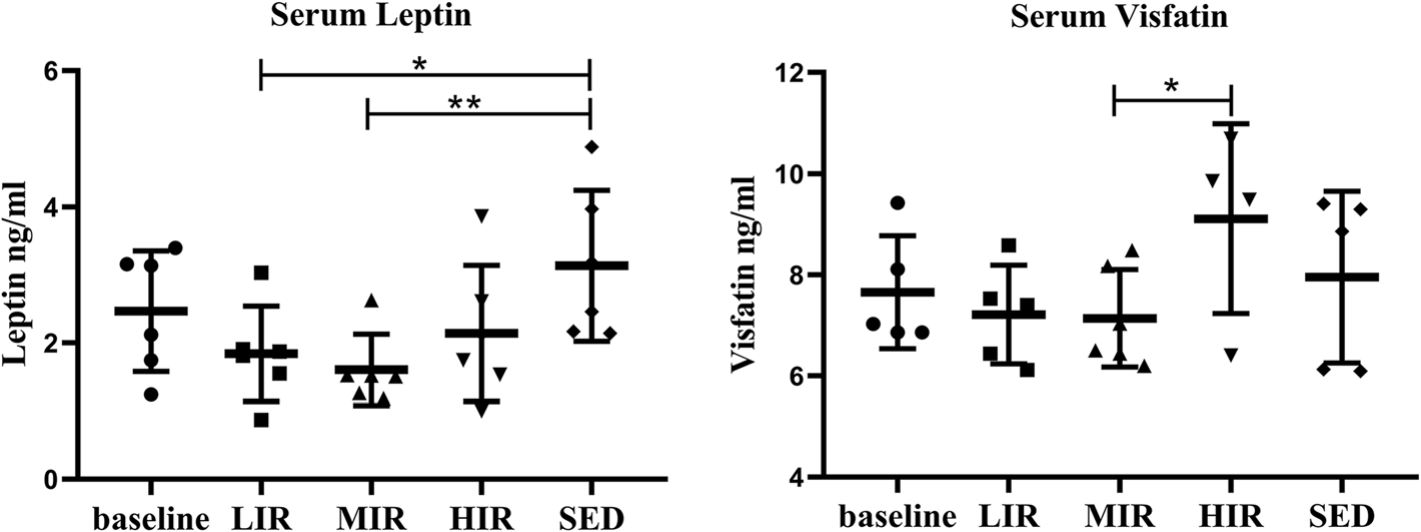
Serum adipokine expression in the five groups: The expression levels of serum leptin and visfatin (data are shown as mean ± SD; *P < 0.05)

Figure 2 presents the serum leptin and visfatin levels in the five groups. For each group, there was no significant difference in either adipokine between the baseline and the 8th week values. Nevertheless, at the 8th week, leptin levels in the LIR (1.84 ± 0.69 ng/ml, *P* = 0.016) and MIR (1.60 ± 0.52 ng/ml, *P* = 0.005) groups were significantly lower than those in the SED group (3.31 ± 1.10 ng/ml). The leptin level was lower in the HIR group (2.14 ± 1.00 ng/ml) than in the SED group, although the difference was not statistically significant. However, the serum visfatin level was significantly lower in the LIR (7.21 ± 0.98 ng/ml) and MIR groups (7.13 ± 0.97 ng/ml) than in the HIR group (9.10 ± 1.87 ng/ml).

### IFP morphometry, histology, and immunohistochemistry

#### The morphometry of IFP adipocytes

The results of the morphometric analysis of IFP adipocytes in the five groups are shown in Table 2. Except for the number of adipocytes and the number of adipocytes per unit area in the SED group (69.22 ± 5.68 vs. 49.89 ± 3.27, *P* = 0.032 and 1002.00 ± 142.35 vs. 722.15 ± 82.07, *P* = 0.03, respectively), there were no significant differences in the groups between the baseline and 8th week values of each parameter. Additionally, for the values of the parameters at the 8th week, no significant differences were found among the four groups.

**Table 2:** The morphometry of adipocytes in IFP

	Number of adipocytes	Total area of adipocytes	Area of single adipocyte	Number of adipocytes per unit area	Adipocyte diameter
Baseline	69.22 (5.68)	0.0645 (0.0038)	0.00095 (0.00027)	1002.00 (142.35)	0.0394 (0.0032)
8th week					
LIR	54.67 (8.33)	0.0668 (0.0030)	0.00129 (0.00026)	791.31 (170.59)	0.0418 (0.0065)
MIR	61.67 (2.91)	0.0652 (0.0024)	0.00110 (0.00007)	892.63 (73.02)	0.0389 (0.0004)
HIR	59.33 (7.46)	0.0665 (0.0009)	0.00110 (0.00005)	858.86 (186.94)	0.0330 (0.0012)
SED	49.89 (3.27)*	0.0640 (0.0026)	0.00131 (0.00019)	722.15 (82.07)*	0.0402 (0.0029)

Values are mean (SD); ∗*P* < 0.05 compared to baseline

#### Cellularity

**Fig. 3:**
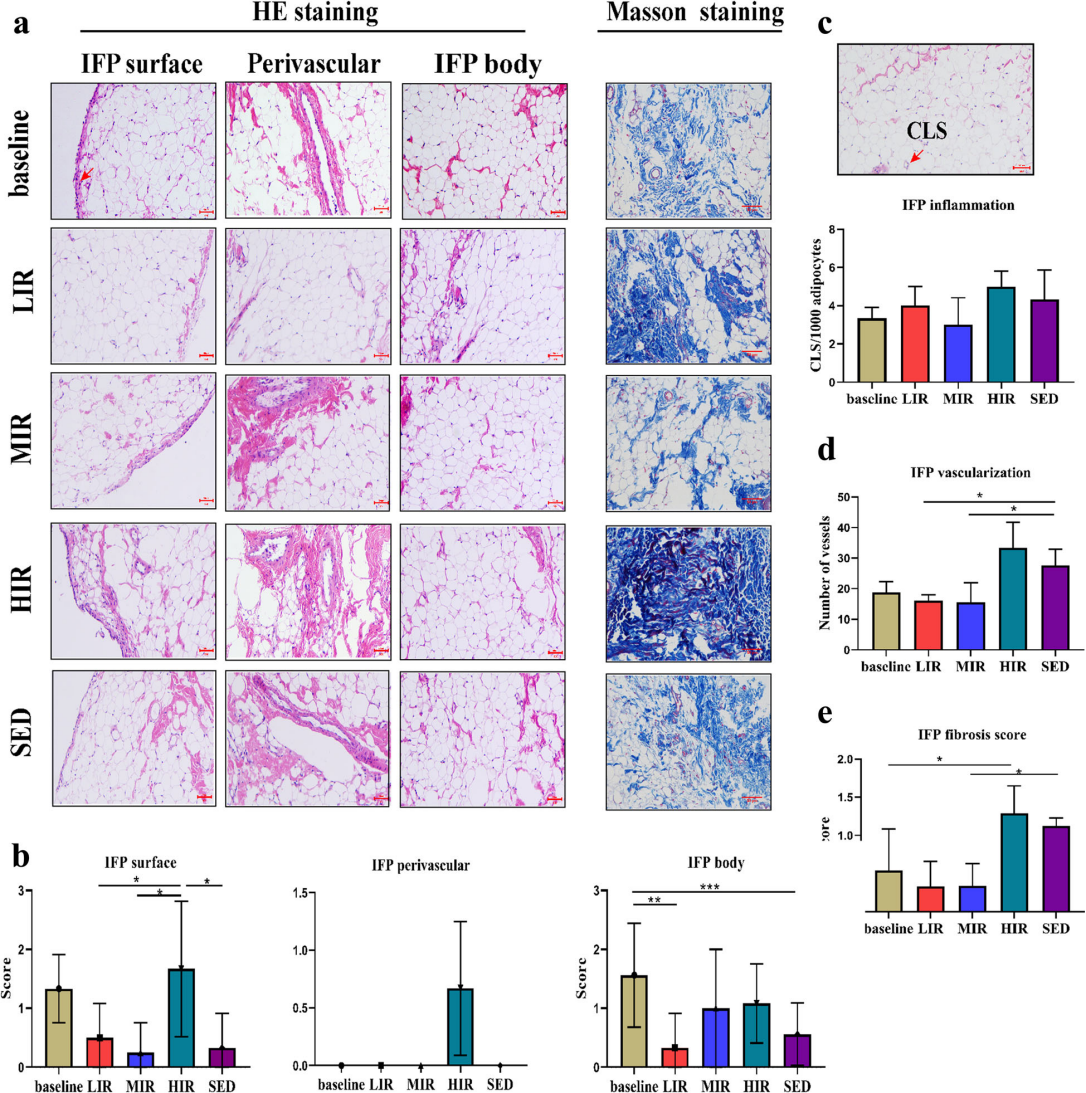
Histological features of the infrapatellar fat pad in the five groups: **a** Representative photographs showing HE staning of the IFP body, perivascular region, and IFP surface( highlighted by the arrow) regions and Masson staining in the baseline, LIR, MIR, HIR and SED groups. **b** Semiquantative evaluation of the cell number in the IFP body, perivascular region and surface. **c** Crown-like structure (highlighted by the arrow) count in the IFP, expressed per 1,000 adipocytes. **d** Semiquantitative evaluation of IFP vascularization. **e** Semiguantitative evaluation of IFP fibrosis. Scale bar represents 50 μm. *P < 0.05; **P < 0.01; ***P < 0.001

Figure 3 presents images of the IFP body, perivascular region, and IFP surface regions (3A), as well as the results of the semiquantitative evaluation of cell number (3B). For the cell number between the baseline and the 8th week, a significant difference was found for the IFP body region in the LIR group (1.56 ± 0.88 vs. 0.33 ± 0.58, *P* = 0.016) and the SED group (1.56 ± 0.88 vs. 0.56 ± 0.53, *P* = 0.006). In contrast, at the 8th week, no significant differences were found in cell number among the four groups for the IFP body region or perivascular region. However, for the IFP surface region, the cell number was significantly higher in the HIR group (1.67 ± 1.15) than in the LIR (1.67 ± 1.15 vs. 0.50 ± 0.58, *P* = 0.049), MIR (1.67 ± 1.15 vs. 0.25 ± 0.50, *P* = 0.023), and SED (1.67 ± 1.15 vs. 0.33 ± 0.58, *P* = 0.040) groups.

#### IFP inflammation

Adipose inflammation in the IFP was assessed using the number of CLS/1000 adipocytes. As shown in Fig. 3C, for each group, no significant difference was found between the numbers at baseline and the 8th week. Similarly, there were no significant differences among the four groups in the values at the 8th week.

#### Vascularization

As shown in Fig. 3D, the vascularity of the IFP in the HIR group was statistically significantly higher (33.31 ± 8.43) at the 8th week than at the baseline (18.83 ± 8.50) (*P* = 0.007), while no significantly differences were found in the other three groups between the baseline and 8th week values. For the 8th week values, a significant lower score was found in the LIR (16.00 ± 2.00, *P* = 0.034) and MIR (15.50 ± 6.44, *P* = 0.013) groups than the SED group (27.58 ± 5.36).

#### Fibrosis

IFP fibrosis was scored using Masson staining. As shown in Fig. 3E, a significant difference was found only in the HIR group between the baseline (0.54 ± 0.54) and 8th week (1.29 ± 0.36) values (*P* = 0.019). However, at the 8th week, a significantly lower score was found in the MIR group (0.34 ± 0.29) (*P* = 0.021) and the LIR group (0.33 ± 0.33) (*P* = 0.020) than the SED group (1.12 ± 0.10).

#### Macrophage infiltration

**Fig. 4:**
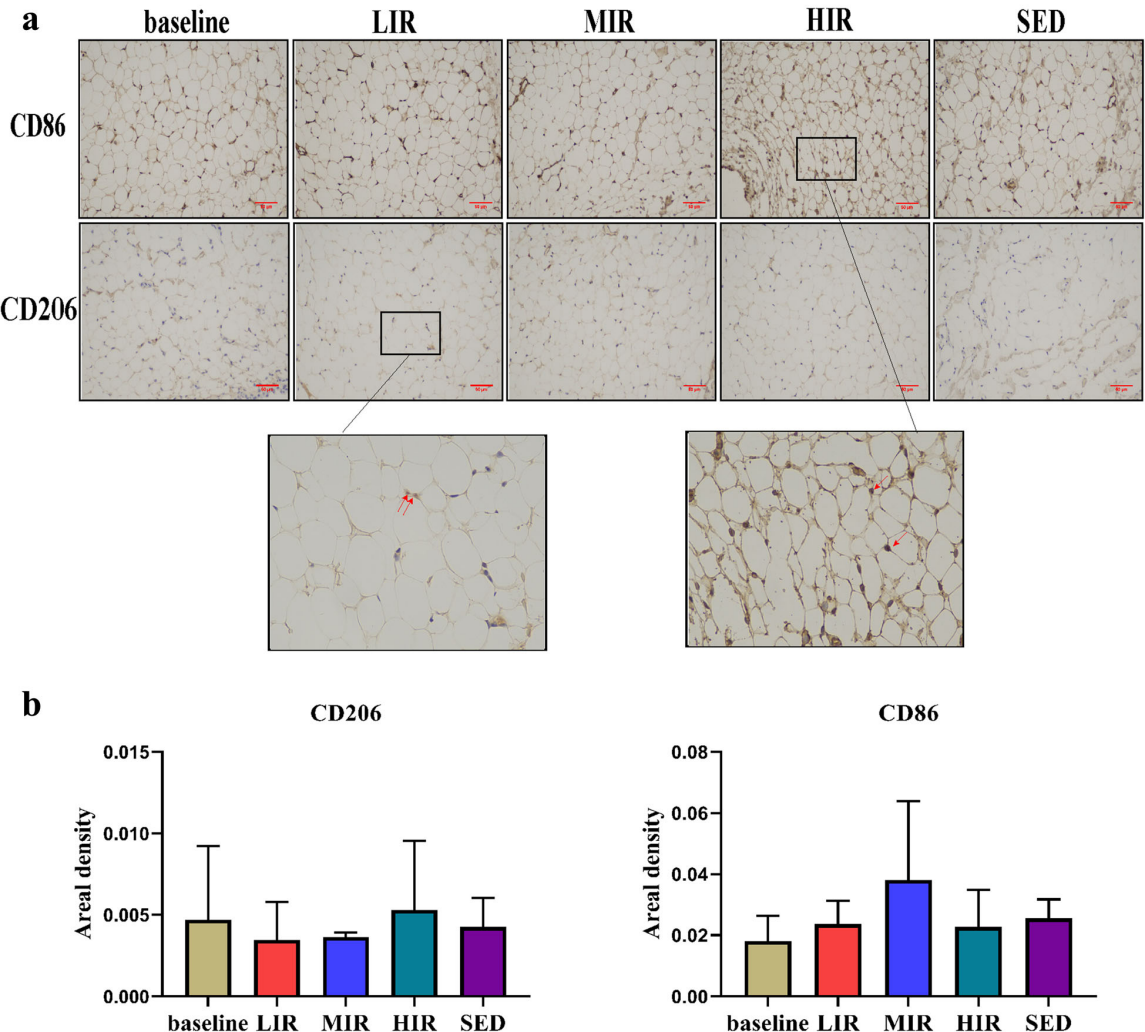
Macrophages in the IFP in the five groups: **a** Representative images of the immunohistochemistry staining. Positive cells are shown in red and indicated with arrows. **b** Statistical analysis. Scale bar represents 50 μm

We evaluated the effects of different intensities of running on CD86 and CD206 adipose tissue macrophages, as determined using immunohistochemical staining. There was no significant difference in any group in the presence of CD86^+^ and CD206^+^ cells between the baseline and 8th week. Similarly, at the 8th week, there were no significant differences among the four groups in the presence of CD86^+^ and CD206^+^ cells (Fig. 4).

### qRT-PCR analysis

**Fig. 5:**
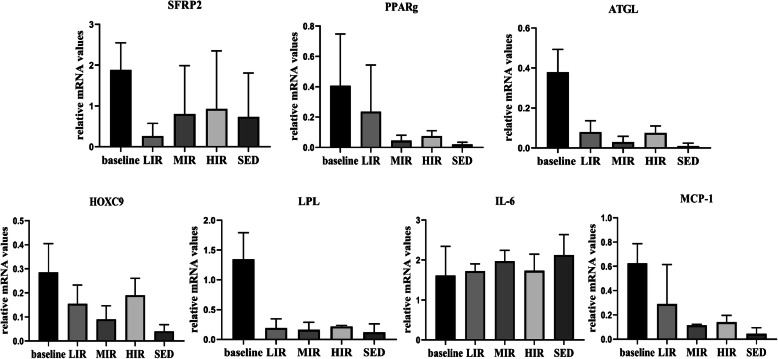
mRNA expression in the IFP in the five groups: The mRNA expression levels of SFRP2, PPARg, ATGL, HOXC9, LPL, IL-6 and MCP-1in the IFP at baseline and at the 8^th^ week in the LIR, MIR, HIR and SED groups(data are shown as the mean ± SD)

Figure 5 presents the mRNA expression results of genes related to lipid metabolism (ATGL, LPL, PPAR-g, FABP4), development (SFRP2 and HoxC9), and inflammation (IL-6, MCP-1) in the IFP of the five groups. For each gene, there was no significant difference in any group between the values at the baseline and 8th week. Similarly, at the 8th week, there were no significant differences among the four groups in the expression of any of the genes.

## Discussion

OA is considered to be a whole-joint disease, and IFP is hypothesized to contribute to the risk of knee OA because of its proinflammatory phenotype in OA joints. Although it was reported that exercise affects IFP remodeling and inflammation [9], it remains unknown how the IFP responds to running at different intensities. Our findings indicate that running has an intensity-dependent effect on IFP remodeling. In contrast to LIR and MIR, HIR led to an increase in the cellularity, fibrosis, and vascularity of the IFP. Furthermore, HIR-induced IFP fibrosis was probably due to mechanical stress, rather than pathological proinflammatory M1/M2 polarization.

The IFP is the local adipose tissue located below the patella, and it plays an important role in maintaining the health of the knee joint. Accumulating lines of evidence indicate that this structure is involved in the development of KOA [4, 16]. Using the same animal model as in the present study, we previously found that treadmill running at low-to-medium-intensity maintains cartilage homeostasis [2]. In the present study, we found that low-to-medium-intensity running led to unchanged or decreased fibrosis, vascularity, and surface cellularity in the IFP, which likely contributed to the maintenance of optimal joint homeostasis. High-intensity running, however, may cause cartilage degradation [7]. This effect of high-intensity running may be at least partially attributable to the substantial increase in IFP fibrosis and vascularization as was found in the present study. These pathological structural changes have been reported in patients with KOA [6].

An intensity-dependent effect of running was also found on the expression of serum adipokines. Low-to-medium intensity running resulted in substantially lower leptin levels, whereas high-intensity running was associated with an increase in the visfatin level. Leptin and visfatin can exert their proinflammatory and procatabolic effects on cartilage [14, 17]. Leptin has been shown to regulate chondrocyte anabolism by inducing the mRNA and protein expression of some proinflammatory or procatabolic genes in vivo, including MMP-9, ADAMTS-4/5, NO, PGE2, IL-6, and IL-8, in vivo [18]. In parallel, visfatin has been shown to affect chondrocytes by inducing the expression of MMPs and reducing the synthesis of matrix components [19, 20]. These findings suggest that, contrary to high-intensity running, low-to-medium intensity running reduces high levels of circulating proinflammatory and procatabolic adipokines, thus benefiting joint health.

As noted earlier, high-intensity running may induce a variety of pathological manifestations in the IFP, among which fibrosis may be the most typical characteristic. Abundant fibrosis has been observed in patients with end-stage KOA [6], and increased fibrosis (accompanying increased structural damage) has been observed in mice fed a high-fat diet [5]. Fibrosis is regarded as a ubiquitous tissue response to an unresolved chronic inflammation, and it is also a reparative process initiated during tissue healing in response to injury and related to the degree of joint damage [21]. IFP fibrosis is triggered by inflammation and/or mechanical stress [5, 21, 22]. A possible explanation for the association of adipose tissue fibrosis with inflammation is that CD86^+^M1 positive macrophages generate proinflammatory signals to shift preadipocytes away from an adipogenic lineage and toward a myofibroblast phenotype [5]. However, in this study, similar inflammatory changes were found in the three running groups, including M1 macrophage polarization, crown-like structures, and the expression of inflammatory-related genes, implying that pathologic proinflammatory M1/M2 polarization likely does not serve as the direct trigger factor for HIR-induced IFP fibrosis.

Mechanical stress is widely recognized as the single most important environmental factor responsible for joint homeostasis [23, 24]. Our previous studies demonstrated that running-induced mechanical stimuli may induce an intensity-dependent effect on bone remodeling and cartilage maintenance [2, 13]. It has been suggested that there is a biomechanical “window” for maintaining optimal joint homeostasis [24]. The findings from the present study clearly indicated that mechanical stress plays a vital role in the remodeling of the IFP. Barboza et al. [5] reported additional evidence, suggesting that obesity-induced IFP fibrosis may occur via physiological signaling mediators, such as mechanical stress, rather than pathologic proinflammatory M1 polarization. Further investigations are warranted to better understand the mechanism underlying the responses of the IFP to various mechanical stresses.

There are several potential limitations to this study. First, the lack of information on the expression of pro-inflammatory or anti-inflammatory cytokines and adipokines in the IFP likely limits our understanding of the intensity-dependent effect of running on the IFP inflammation phenotype. Moreover, the immunohistochemical assessment only included M1 and M2 macrophages. Other immune cells such as T cells could also contribute to inflammation of the IFP. In the future, flow cytometry should be used to identify various immune cell types to better understand the origin of IFP inflammation.

In summary, in the present study, the effects of running at different intensities on the IFP were examined. The results indicated that the effect of running on IFP remodeling is intensity dependent. In contrast to LIR and MIR, HIR increased the fibrosis and vascularity of the IFP. Additionally, HIR-induced IFP fibrosis was probably due to mechanical stress, rather than pathological proinflammatory M1/M2 polarization.

## Data Availability

The necessary data were provided to support the assumption of this study (data will be made available on demand).

## Abbreviations

SED: Sedentary
LIR: Low-intensity running
MIR: Medium-intensity running
HIR: High-intensity running
IFP: Infrapatellar fat pad
KOA: Knee osteoarthritis
SAT: Subcutaneous adipose tissue
IL-6: Interleukin-6
TNF: Tumor necrosis factor
HE: Hematoxylin-eosin
CLS: Crown-like structures
IHC: Immunohistochemistry
ELISA: Enzyme-linked immunosorbent assay
ANOVA: A one-way analysis of variance
SD: Standard deviation

## References

[1] Bastiaansen-Jenniskens YM, Clockaerts S, Feijt C, Zuurmond AM, Stojanovic-Susulic V, Bridts C, de Clerck L, DeGroot J, Verhaar JAN, Kloppenburg M, van Osch GJVM (2012). Infrapatellar fat pad of patients with end-stage osteoarthritis inhibits catabolic mediators in cartilage. Ann Rheum Dis.

[2] Ni GX, Liu SY, Lei L, Li Z, Zhou YZ, Zhan LQ (2013). Intensity-dependent effect of treadmill running on knee articular cartilage in a rat model. Biomed Res Int.

[3] Eymard F, Chevalier X (2016). Inflammation of the infrapatellar fat pad. Joint Bone Spine.

[4] Zeng N, Yan Z-P, Chen X-Y, Ni G-X (2020). Infrapatellar fat pad and knee osteoarthritis. Aging Dis.

[5] Barboza E, Hudson J, Chang WP, Kovats S, Towner RA, Silasi-Mansat R, Lupu F, Kent C, Griffin TM (2017). Profibrotic infrapatellar fat pad remodeling without M1 macrophage polarization precedes knee osteoarthritis in mice with diet-induced obesity. Arthritis Rheum.

[6] Favero M, El-Hadi H, Belluzzi E, Granzotto M, Porzionato A, Sarasin G (2017). Infrapatellar fat pad features in osteoarthritis: a histopathological and molecular study. Rheumatology (Oxford).

[7] Gessel T, Harrast MA (2019). Running dose and risk of developing lower-extremity osteoarthritis. Curr Sports Med Rep.

[8] Bohnsack M, Hurschler C, Demirtas T, Rühmann O, Stukenborg-Colsman C, Wirth CJ (2005). Infrapatellar fat pad pressure and volume changes of the anterior compartment during knee motion: possible clinical consequences to the anterior knee pain syndrome. Knee Surg Sports Traumatol Arthrosc.

[9] Barboza EKT, Hudson J, Kovats S, Griffin TM (2017). Exercise induces transient inflammatory and pro-fibrotic remodeling the infrapatellar fat pad. Osteoarthr Cartil.

[10] Timmins KA, Leech RD, Batt ME, Edwards KL (2017). Running and knee osteoarthritis: a systematic review and meta-analysis. Am J Sports Med.

[11] Rios JL, Boldt KR, Mather JW, Seerattan RA, Hart DA, Herzog W (2018). Quantifying the effects of different treadmill training speeds and durations on the health of rat knee joints. Sports Med Open.

[12] Appleton CT (2018). Osteoarthritis year in review 2017: biology. Osteoarthr Cartil.

[13] Li Z, Liu SY, Xu L, Xu SY, Ni GX (2017). Effects of treadmill running with different intensity on rat subchondral bone. Sci Rep.

[14] Warmink K, Kozijn AE, Bobeldijk I, Stoop R, Weinans H, Korthagen NM (2020). High-fat feeding primes the mouse knee joint to develop osteoarthritis and pathologic infrapatellar fat pad changes after surgically induced injury. Osteoarthr Cartil.

[15] Inomata K, Tsuji K (2019). Time course analyses of structural changes in the infrapatellar fat pad and synovial membrane during inflammation-induced persistent pain development in rat knee joint. BMC Musculoskelet Disord.

[16] Ioan-Facsinay A, Kloppenburg M (2013). An emerging player in knee osteoarthritis: the infrapatellar fat pad. Arthritis Res Ther.

[17] Ouchi N, Parker JL, Lugus JJ, Walsh K (2011). Adipokines in inflammation and metabolic disease. Nat Rev Immunol.

[18] Belluzzi E, El Hadi H, Granzotto M, Rossato M, Ramonda R, Macchi V (2017). Systemic and local adipose tissue in knee osteoarthritis. J Cell Physiol.

[19] Gómez R, Conde J, Scotece M, Gómez-Reino JJ, Lago F, Gualillo O (2011). What’s new in our understanding of the role of adipokines in rheumatic diseases?. Nat Rev Rheumatol.

[20] Yammani RR, Loeser RF (2012). Extracellular nicotinamide phosphoribosyltransferase (NAMPT/visfatin) inhibits insulin-like growth factor-1 signaling and proteoglycan synthesis in human articular chondrocytes. Arthritis Res Ther.

[21] Ioan-Facsinay A, Kloppenburg M (2017). Osteoarthritis: Inflammation and fibrosis in adipose tissue of osteoarthritic joints. Nat Rev Rheumatol.

[22] Mack M (2018). Inflammation and fibrosis. Matrix Biol.

[23] Alentorn-Geli E, Samuelsson K, Musahl V, Green CL, Bhandari M, Karlsson J (2017). The association of recreational and competitive running with hip and knee osteoarthritis: a systematic review and meta-analysis. J Orthop Sports Phys Ther.

[24] Ni GX (2016). Development and prevention of running-related osteoarthritis. Curr Sports Med Rep.

